# Phytoremediation potential and copper uptake kinetics of Philippine bamboo species in copper contaminated substrate

**DOI:** 10.1016/j.heliyon.2019.e02440

**Published:** 2019-09-17

**Authors:** Jennivee Chua, Jessa Marie Banua, Ivan Arcilla, Aileen Orbecido, Maria Ellenita de Castro, Nadine Ledesma, Custer Deocaris, Cynthia Madrazo, Lawrence Belo

**Affiliations:** a Chemical Engineering Department, Gokongwei College of Engineering, De La Salle University, 2401 Taft Avenue, Manila, 0922, Philippines; b Department of Forest Biological Sciences, College of Forestry and Natural Resources, University of the Philippines Los Baños, Philippines; c College of Agriculture, University of Rizal System Main Campus, Sampaloc, Tanay, Rizal, Philippines; d Technological Institute of the Philippines, Quezon City, Philippines; e Commission on Higher Education, Quezon City, Philippines

**Keywords:** Environmental science, Phytoremediation, Bamboo, Copper, Kinetics, Uptake, Michaelis–Menten

## Abstract

The phytoremediation potential of three bamboo species, *i.e. Bambusa merilliana*, *Bambusa blumeana*, and *Dendrocalamus asper*, were evaluated for their total Cu uptake ability in hydroponics. *Dendrocalamus asper* proved to be the most efficient in terms of Cu phytoremediating potential with a constant positive uptake of 80 μM in a contaminated substrate and a bioconcentration factor of 50.57. Copper accumulation was found to concentrate the most in the roots compared to the amount translocated in the shoots. Analysis of the Cu uptake of *D. asper* roots at varying concentrations of Cu contamination (40, 80 and 120 μM) allowed for the fitting of the kinetics of Cu uptake and removal with existing kinetic models. The rate of copper removal per mass of plant was the best for the 0^th^ order model in the 80 μM solution with an R^2^ of 0.954 and rate constant of 3.136 mg-kg^−1^d^−1^. The accumulation of Cu within the roots on day 7 (7d) followed the Michaelis–Menten model with an R^2^ of 0.970. The Michaelis–Menten constant (K_M_) was 4.87 mg/L and maximum accumulation velocity (Vmax) was 66.26 mg Cu-kg^−1^-day^−1^. Results suggest that *D. asper* is a potential hyperaccumulator plant that can be used in clean-up of domestic and industrial wastes present along river systems in the Philippines.

## 1 Introduction

Rapid industrialization and the accelerated growth of industrial activities have brought about demand for various products that contain toxic and hazardous chemicals such as heavy metals (HMs) or potentially toxic elements (PTEs). In the Philippines, industries like tanning and jewelry have increased their production capacities leading to the inevitable consequences of metal waste, particularly copper. Copper is an important engineering material, but it is regarded as one of the earliest known PTEs (Shrivastava, 2009). Copper pollution is considered deleterious to human health and the environment because of its nonbiodegradability, biomagnification potential, and persistence in the environment (Lone et al., 2008; Shrivastava, 2009). Copper contamination in water and soil is widespread in the Philippines due to the activities of the mining, tanning and jewelry industries. For instance, in samples taken from rivers in Bulacan, Philippines, 3.14 mg/L of Cu was detected, which is in large excess of the 0.02 mg/L acceptable limit set by the country's Department of Natural Resources – Environmental Management Bureau (DENR-EMB) for class C waters (ADB, 2009; Belo et al., 2018; DENR, 2016). While copper is an essential element for some of the biological processes of living organisms, excessive amounts can be toxic (Lange et al., 2017). Excessive exposure of humans to copper can lead to headaches, vertigo and impotence while ingestion can cause anemia, jaundice and even death (ATSDR, 2004). For plants, optimal growth conditions are achieved with 2–20 mg/L Cu; higher Cu concentrations may interrupt cell functions, inhibit growth and cause an imbalance in nutrient uptake (Chen et al., 2015a; Lone et al., 2008; Zappala et al., 2014). Because of the risks posed by PTE contamination of the environment, the pursuit for cost-effective and sustainable remediation technologies to remove/reduce contaminants from the environment is imperative (Cheng, 2003; Fulekar, 2016; Pachon, 2011; Tangahu et al., 2011). Current removal methods, such as pump-and-treat, thermal desorption, soil-vapor extraction, *ex situ* stabilization are either energy intensive or too expensive (Pachon, 2011; Sharma et al., 2016). Furthermore, some of the treatment methods may involve utilization of hazardous chemicals that could pose additional threats to the ecosystem (Wuana and Okieimen, 2011). Therefore, the need to utilize environmentally sustainable remediation techniques such as phytoremediation has become imperative. Phytoremediation is an *in situ* remediation technique that utilizes the unique ability of some plants to hyperaccumulate organic and toxic metal contaminants from the water, soil or air (Ali et al., 2013; Baker et al., 1999; Cheng, 2003; Dickinson et al., 2009; Sharma et al., 2016). The tolerance of plants to contaminants arise from different defense mechanisms such as toxicity reduction, contaminant storage in the vacuole and avoidance mechanisms (Hall, 2002). Compared to other remediation methods, phytoremediation is a cost-effective approach as it eliminates the need for high capital in equipment (Wang et al., 2002) and is solar-powered as it utilizes energy directly from the sun (Pachon, 2011). Its economic importance can be realized through phytomining or phytoextraction approach in which the contaminants are directly harvested from the collected aboveground plant tissues after uprooting (Chaney and Mahoney, 2014). This agronomic approach takes into account the ability of the plant to intoxicate the PTEs present in the soil and possibly concentrate them in the soil layer so as not to affect the growth of the existing standing crops. However, phytomining technology is still at its infancy and several factors must be taken into consideration prior to its application more importantly the recovery rate of specific toxic elements in the plant biomass (Chaney and Mahoney, 2014).

It should further be noted that phytoremediation is not a cure-all approach to rehabilitation and its potential for success largely depend on the characteristics of the plant to be utilized and the contaminant present in the ecosystem concerned. And though several plant species has already been tested for their hyperaccumulating potentials, majority are not yet considered successful due to the inherent slow-growing ability, endemism and short stature (Prasad and Freitas, 2003). There were also records that some identified hyperaccumulators have short root system which could possibly limit their absorbing capacity. Therefore, applying phytoremediation to actual contaminated sites requires a detailed and thorough study on the contaminant uptake of the plant as well as kinetic studies to determine the time and number of plants to be introduced for the remediation.

Bamboo is a member of grass family, *Graminae* (*Poaceae*) that has the ability to survive even in the most marginalized soil and climatic conditions (Rojo et al., 2000; Roxas, 2012). Unlike other grass species, however, bamboos are sturdy due to the presence of huge amount of sclerenchyma cells, usually with hollow culm, complex rhizome and branched system (Rojo et al., 2000). An estimated 1000 species of bamboo species belonging to 80 genera are growing in various parts of the world (Yeasmin et al., 2015). In the Philippines alone, over 70 species of bamboo are growing mostly in the thickest of the forest and along river systems (Roxas, 2012; Virtucio et al., 2007). They are economically important in the country due to their versatility are commonly used in construction, furniture, basketry, and decorative articles (Espiloy et al., 2007; Rojo et al., 2000; Roxas, 2012; Virtucio et al., 2007). As a potential hyperaccumulator, bamboo is a fast-growing species that meets the demands for high biomass production which could facilitate the immediate removal of toxic metals from the water or soil. It will also require very minimal production requirements, hence less expensive to grow and maintain. Apart from evaluating the phytoremediation potential of Philippine bamboos, the initiative of using bamboos will support the Philippines Executive Order 879, s. 2010 directing the use of bamboo for at least 25% of desks in public primary and secondary schools; as well as Executive Order 193, s. 2015 also known as the expanded National Greening Program directing reforestation and reintroduction of biodiversity.

Plants that produce a large root biomass and fast-growing such as bamboo could be an appropriate alternative for phytoremediation (Gerhardt et al., 2009). For instance, *Phyllostachys pubescens*, a bamboo species most common in temperate Asia, has been reported to have an underground biomass concentration that of about 85.5–94.0 ton ha^−1^, which is higher than most forest species (Emamverdian et al., 2018). Most published studies on the phytoremediation potential of bamboo have focused on *P. pubescens* under both hydroponic and natural soil conditions. A study (Bian et al., 2017) likewise reported its high accumulation abilities in soils contaminated with copper, cadmium, and zinc. Other reports also confirmed the potential of the said species to accumulate copper, zinc, cadmium and lead in the roots, shoots, and leaves in hydroponic set-ups (Bin et al., 2017; Chen et al., 2014, 2015a, 2015b; Li et al., 2015; Zhang et al., 2010).

The use of other bamboo species as phytoremediators in heavy metal-contaminated soil and wastewater have also been reported for copper using *Semiarundinaria fastuosa*, *Phyllostachys viridis*, and *Phyllostachys viridis* “sulfurea” (Arfi et al., 2009) and for chromium using several species of *Dendrocalamus* and *Bambusa* (Were et al., 2017). There have yet no published studies that the authors are aware on the phytoremediation potential of bamboo species native to the Philippines. Thus, for the present study, three native bamboo species, *Bambusa blumeana, Bambusa merilliana* and *Dendrocalamus asper*, whose Cu removal potential has not been reported, were tested using contaminated hydroponic solutions. The bamboo were grown using hydroponics to provide the optimal nutrient conditions to the plants with no possibility of interference from soil-borne biotic and abiotic factors. Moreover, the nutrient supply can be easily controlled and adapted over time as needed, which is important for the kinetic measurements. It could also be useful in determining the possibility of using bamboo in the treatment of wastewater which is a very common pollutant in the Philippines.

The kinetics of both contaminant removal and contaminant accumulation within the plant were also studied and fitted to existing models from the literature (Arcilla et al., 2017a). Determination of the kinetic models in the study will allow for the application of the plant in phytoremediation as knowledge of the kinetic parameters will determine the planting set up for application in remediation.

## 2 Materials and methods

### 2.1 Plant materials

The three species of bamboo were purchased from a commercial nursery in Victoria, Laguna, Philippines. *B. merilliana* and *D. asper* culm cuttings were six months old while the *B. blumeana* were only two-and-a half months old. The plants were grown in the greenhouse of Biology Department, De La Salle University. One week of stabilization was allowed for the potted plants to acclimatize to the new conditions.

### 2.2 Hydroponics setup

After acclimatizing for one week, the plants were then uprooted from the soil media, washed with tap water and transferred in the hydroponics setup. The nutrient solution used for this experiment was the Simple Nutrient Addition Program (SNAP) solution developed by the Soil Science Department of the University of the Philippines Los Baños (UPLB). The hydroponics setup from SNAP uses two commercially available formulations: SNAP A and SNAP B. The solution was prepared in accordance with manufacturer's instructions: 25mL of SNAP A was added to 10L of distilled water. After mixing, 25mL of SNAP B was added. Each pot contained 1L of the hydroponics solution. The plants were allowed to grow in the hydroponics solution for another week prior to the Cu contamination experiment.

For the entire duration of the experiment, the bamboo plants were exposed to natural outdoor light supplemented with eight 54-W high-output fluorescent lamps for 14 hours daily. Light intensity was 700 μmol m^−2^·s^−1^ on average near the leaf surface. Average day/night temperatures were 32 °C/27 °C.

### 2.3 Experimental runs

The following sections discuss in detail the procedures carried out in the conduct of the three experimental runs in the study: (1) determination of the best bamboo species for phytoremediation; (2) determination of the best concentration for phytoremediation of the selected species; and (3) kinetic modeling of Cu removal and uptake.

#### 2.3.1 Determination of the best bamboo species for phytoremediation

One week after their transfer into the hydroponics setup, the nutrient solution was spiked with 80 μM Cu as CuSO_4_ – an amount that was shown to cause no toxic effects on plants (N. V. Prasad, 2004). A total of 30 plants were used (one control for each species and triplicate samples for each species per week of destructive sampling). A week later, triplicate samples of each bamboo species were uprooted and weighed. Sampling of the nutrient solution from all treatments (bamboo species) were obtained and analyzed for Cu concentration using Flame Atomic Absorption Spectrophotometry (AAS), Shimadzu AA-6300. Sampling of nutrient solutions were for three consecutive weeks to determine the rate of Cu removal in the solution. Each sample had a volume of 5mL to minimize volume reduction of the solution. The bamboo species whose nutrient solution had the highest Cu removal was selected for the succeeding phases of the study.

#### 2.3.2 Translocation of copper in *Dendrocalamus asper*

From the results obtained from Section 2.3.1, *D. asper* was determined to be the best species for Cu removal from the hydroponic solution. The next phase of the study involved determining the translocation of Cu within the different plant parts of *D. asper* within a three-week period. The plant samples were acid-digested per plant part (roots, culm, branches, and leaves) to determine which part had the largest concentration of Cu to aid in the determination of the translocation factor (TF). Acid digestion of the plant samples was carried out using the method suggested by Pequerul et al. (1993). The plant samples were oven dried, grounded and digested using the modified USEPA method 3050B in *aqua regia* prepared from 15M HNO_3_ and 12M HCl in the molar ratio of 1:3. The solution was left overnight for the powdered samples to be completely immersed in the acid mixture, which resulted in a rust like color. After 12 hours, the flask was heated on a hot plate set at around 160 °C to digest the samples and allow excess acid to evaporate. Once the solution turned clear and the excess acid evaporated, the samples were taken off the plate and allowed to cool for a few minutes. After cooling, two (2) milliliters of 30%v H_2_O_2_ was added, and the sample was set back on the hot plate, allowing the solution to boil once more. Once boiling, the sample was taken off the hot plate and allowed to cool for a few minutes. After cooling, twenty (20) milliliters of distilled water was added and the sample was placed back on the hot plate and heated until boiling one last time.

The resulting solution was filtered into a fifty (50) milliliter volumetric flask and diluted to mark with distilled water. The samples were transferred into glass vials and tested for Copper (Cu) concentration using an Atomic Absorption Spectrophotometer (Pequerul et al., 1993). To neutralize the obtained solutions, 1 M NaOH was added at 3:10 v/v ratio prior to AAS for Cu concentration determination. Eq. (1) was used in solving for the TF.

(1) $$TF=\frac{C_{aerial}}{C_{roots}}$$

#### 2.3.3 Determination of the highest concentration for phytoremediation

Phase 1 revealed that *D. asper* was the best species for copper absorption, hence it was selected as the bamboo species to be used in the next phase of the study, that is, determination of the highest concentration of Cu that can be accumulated in its plant tissues. A new set of *D. asper* plants (30 plants) were obtained from the same source. The plants were conditioned as in Sections 2.1 and 2.2 and set up as triplicate samples. After seven days, the solutions were spiked with Cu to create varying concentrations (40 and 120 μM). A week later, triplicate samples of the whole plants were uprooted, oven-dried, ground and acid-digested to determine the Cu concentration. The sampling was done for another three consecutive weeks.

#### 2.3.4 Kinetic modelling of Cu removal and uptake

Kinetic modelling was carried out to determine which of the kinetic models in literature best fit the data obtained in the study. The data used for these models were either based on the contaminant removal from the nutrient solution or from the contaminant uptake within the plant analyzed through AAS. Table 1 is a summary of the kinetic models used for phytoremediation of various contaminants. Table 2 lists the assumptions coupled with the kinetic models.

**Table 1:** Summary of kinetic models applied in various phytoremediation studies.

Plant	Contaminant	Duration	Model	Rate Constants	Reference
Cottonwood, Willow, Eucalyptus	Perchlorate	52 days	0^th^ & 1^st^ Order Removal	*k* = 0.86–0.95 mg/L/d	(Nzengung, 1998)
Vetiver grass	Prometryn	67 days	1^st^ Order Removal	*k* = 1.807 d ^−1^	(Sun et al., 2016)
*E. crassipes*	Mercury	7 & 14 days	Michaelis–Menten	*V*_*max*_ = 7.184–13.123 mg/L/h	(Narang et al., 2011)
*E. crassipes*	Cyanide	4 hours	Michaelis–Menten	Not Reported	(Ebel et al., 2007)
*S. pectinate*, *C. Stricta*	Arsenic	4 hours	Michaelis–Menten	*K*_*M*_ = 21.3–74.4 mg/L *V*_*max*_ = 24.5–46.5 mg/L/h	(Rofkar and Dwyer, 2011)
*M. guttatus*	Copper	2 hours	Michaelis–Menten	*K*_*M*_ = 2.22–21.38 μM *V*_*max*_ = 0.67–5.05 μM g^−1^ h^−1^	(Strange and Macnair, 1991)

Note: k = rate constant; KM = Michaelis–Menten Constant, Vmax = maximum reaction velocity.

**Table 2:** Assumptions in kinetic models used in phytoremediation.

Model	Contaminant	Measured Parameter	Assumption
Rate of Disappearance	Perchlorate, Cu & Zn	Contaminant concentration of the solution at time, *t*	Constant solution volume
Michaelis–Menten	Cyanide	Contaminant concentration of the solution at time, *t*, converted to mass CN loss/mass of the plant	Constant solution volume
Michaelis–Menten	As, Hg and Cu	Contaminant accumulation within the roots at time, *t*	Constant solution concentration

Combined with the data obtained from AAS of the samples at 80 μM, the Bioconcentration Factor (BCF) at the last day (Day 21) was obtained for each of the initial contaminant concentrations. Eq. (2) shows the formula for the BCF:

(2) $$BCF=\frac{C_{plant}}{C_{initial}}$$

##### 2.3.4.1 Kinetic models of contaminant removal

Previous studies (Arcilla et al., 2017a; Chen et al., 2015a; Cui and Schröder, 2016; Strange and Macnair, 1991) provides kinetics or rates of Cu removal as shown in Eq. (3). Due to the large root system and the significant volume uptake of bamboo, there is a possibility of Cu concentration change due to decreasing solution volume rather than Cu removal. A more accurate account of Cu removal can be obtained by expressing the Cu uptake as a function of the plant mass leading to the rate equation shown in Eq. (4). Expressing the contaminant removal as a function of the plant mass, which can be readily integrated to form a linear equation led to Eq. (5) which assumes negligible solution evaporation and Cu loss accounted by uptake of the plant. The general differential equation is shown in Eq. (3) where the rate of contaminant disappearance is proportional to the contaminant concentration in the media.

(3) $$-\frac{dC_{S}}{dt}=k{C_{S}}^{n}$$

where ***C***_***S***_ is the concentration of the contaminant in the solution at time ***t***, ***k*** is the rate constant whose units depend on the order or reaction, ***n***. The order of reaction in literature is often either 0 or 1.

(4) $$\frac{dC_{L}}{dt}=k{C_{S}}^{n}$$

where ***C***_***L***_ is the mass of Cu loss from the media per mass of the plant, ***v***_***i***_ is the initial volume of the solution, ***m***_***i***_ is the initial mass of Cu in the media, ***w*** is the mass of the plant, and ***a*** and ***b*** are the slopes and intercept, respectively. Using Eq. (4) where ***n*** = 1 (or any order except 0) requires expressing ***C***_***S***_ as a function of ***C***_***L***_ as shown in Eq. (5).

(5) $$\int_{0}^{C_{L}}\frac{dC_{L}}{\frac{m_{i}}{w}-C_{L}}=\int_{0}^{t}\frac{k}{\frac{v_{i}}{w}-\left(at+b\right)}dt$$

##### 2.3.4.2 Kinetic model in contaminant uptake: Michaelis–Menten

Another kinetic model utilized to characterize contaminant uptake by the plant is the Michaelis–Menten equation (Arcilla et al., 2017a; Narang et al., 2011). The concept originated from the enzymatic reactions converting organic compounds and other metabolic reactions to less toxic products. The reaction velocity for PTEs is expressed as the rate of contaminant accumulation (amount of Cu) within the plant per mass of the plant dry weight and is related to the contaminant concentration in the solution as shown in Eq. (6).

(6) $$V=\frac{V_{\max}C_{i}}{K_{M}+C_{i}}=\frac{dC_{P}}{dt}$$

where ***V*** or the reaction velocity is expressed as the rate of product formation in enzymatic reactions; for phytoremediation, particularly in this experiment utilizing hydroponics, the product formation expressed as the rate of contaminant accumulation in the roots per dry weight of the plant, ***C***_***P***_. ***K***_***M***_ is the Michaelis–Menten constant and ***V***_***max***_ is the maximum reaction velocity. The assumption for using the model is a constant solution concentration. The concentration of the solution is either maintained by renewing the solution at certain time intervals or by conducting the experiment in a short duration such that the final concentration may be verified to have insignificant changes with respect to the initial concentration.

Table 3 shows the linearized forms of the differential equations. The overall methodology accompanied by the variables and analyses methods are shown in Table 4.

**Table 3:** Equations for kinetic modelling.

Model	Order	Linearized Form
$\frac{dC_{S}}{dt}=-kC_{S}^{n}$	0	$C_{S}=kt+C_{i}$
	1	$\ln C_{S}=-kt-\ln C_{i}$
$\frac{dC_{L}}{dt}=kC_{S}^{n}$	0	$C_{L}=kt$
	1	$\ln\left(\frac{\frac{m_{i}}{w}-C_{L}}{\frac{m_{i}}{w}}\right)=\ln\left(\frac{\frac{v_{i}}{w}-\left(at+b\right)}{\frac{v_{i}}{w}-b}\right)\cdot k$
$\frac{dC_{P}}{dt}=\frac{V_{\max}C_{i}}{K_{M}+C_{i}}$	N/A	$\frac{t}{C_{P}}=\frac{K_{M}}{V_{\max}C_{i}}+\frac{1}{V_{\max}}$

**Table 4:** Summary of the methodology and analyses.

Objective	Variables	Constant Parameters	Measured Parameters	Analysis
Best Bamboo Species	Bamboo Species: a. *B. merilliana* b. *B. blumeana* c. *D. asper*	Initial Cu Concentration in Solution: 80 μM	Concentration of the Solution	AAS
Cu translocation	Plant Parts a. Roots b. Culm c. Branches d. Leaves	Bamboo Species: *D. asper* Initial Cu Concentration in Solution: 80 μM	Concentration within the plant parts	Acid digestion and AAS
Best initial concentration for phytoremediation	Initial Cu Concentration a. Control b. 40 μM c. 80 μM d. 120 μM	Bamboo Species: *D. asper*	Concentration of the solution and within the roots	Acid digestion and AAS
Kinetic Modelling	Kinetic Models	Bamboo Species: *D. asper*	Concentration of the solution and within the roots	(Table 3)

#### 2.3.5 Statistical analysis

Design Expert ver. 10.0.7 was used in analyzing the data points in Phase 1 of the study: bamboo species selection (*B. bluemana, B. merilliana, D. asper*), and Phase 2 of the study: varying concentration level of Cu contaminant using the species selected in Phase 1. One-way ANOVA was used in determining the significance level of the data.

## 3 Results and discussion

### 3.1 Bamboo species selection

**Fig. 1:**
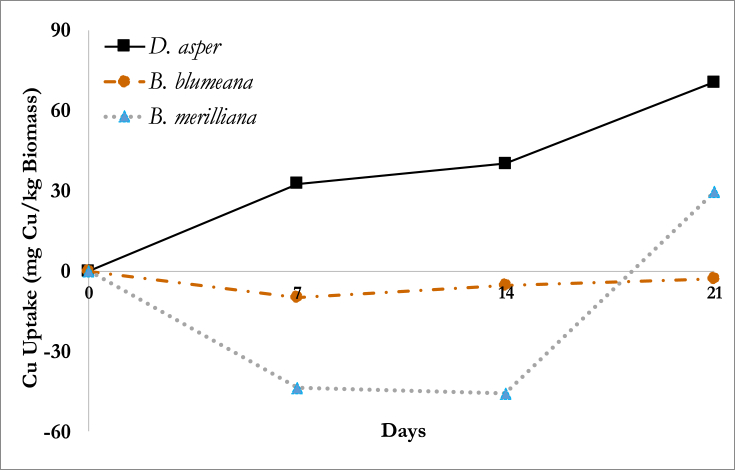
Copper uptake of *Dendrocalamus asper*, *Bambusa blumeana*, and *Bambusa merilliana*.

Fig. 1 shows the amount of Cu taken up by the plants from the nutrient solution per dry plant mass. The amount of Cu taken up by the plants were derived from the Cu loss from the nutrient solution. Briefly, the Cu loss from the nutrient solution was computed as follows: Volume of initial solution (1L) x concentration of solution (20μM) – volume of the solution at time of plant uproot (Day 7, 14 or 21) x concentration of the remaining solution. It can be observed that 7d after the addition of Cu, only the *D. asper* exhibited a consistently positive Cu uptake while *B. blumeana* and *B. merilliana* lie in the negative plane indicating that the Cu concentration in the plant tissues may have been too low to be detected. However, from 14d to 21d, an increase in uptake was observed for the two species *B. bluemana* and *B. merilliana*. Release of the contaminant may be due the efflux of the ions or through ion leakage (Hall, 2002). The copper released was possibly from the initial Cu present in *B. blumeana* and *B. merilliana* prior to the addition of the Cu contaminant. Only the *D. asper* was tested for the initial Cu content within the plant, and analysis of its roots showed a Cu concentration of 60.81 mg/L. This suggests that for bamboo, initial response maybe due to the uptake the Cu however, should there be cell damage or after reaching its threshold level, Cu ions may simply leak out. Nedelkoska and Doran (2000) suggested that for the first few hours upon contamination, Cu may adsorb to the root surface without affecting plant viability (Nedelkoska and Doran, 2000). Because of the possible initial adsorption, the root cells may have been damaged, thus causing the ion leakage. The uptake of the *B. merilliana* may be an indication of slow cell repair after the damage. According to Emamverdian et al. (2015), metallothioneins are the active substances that maintain Cu homeostasis in plants (A. Emamverdian et al., 2015). This can explain the sudden uptake of copper on 21d. After 14 days of copper release, the metallothioneins possibly allowed the transport of copper into the plant to achieve homeostasis (A. Emamverdian et al., 2015).

### 3.2 Translocation of copper

**Fig. 2:**
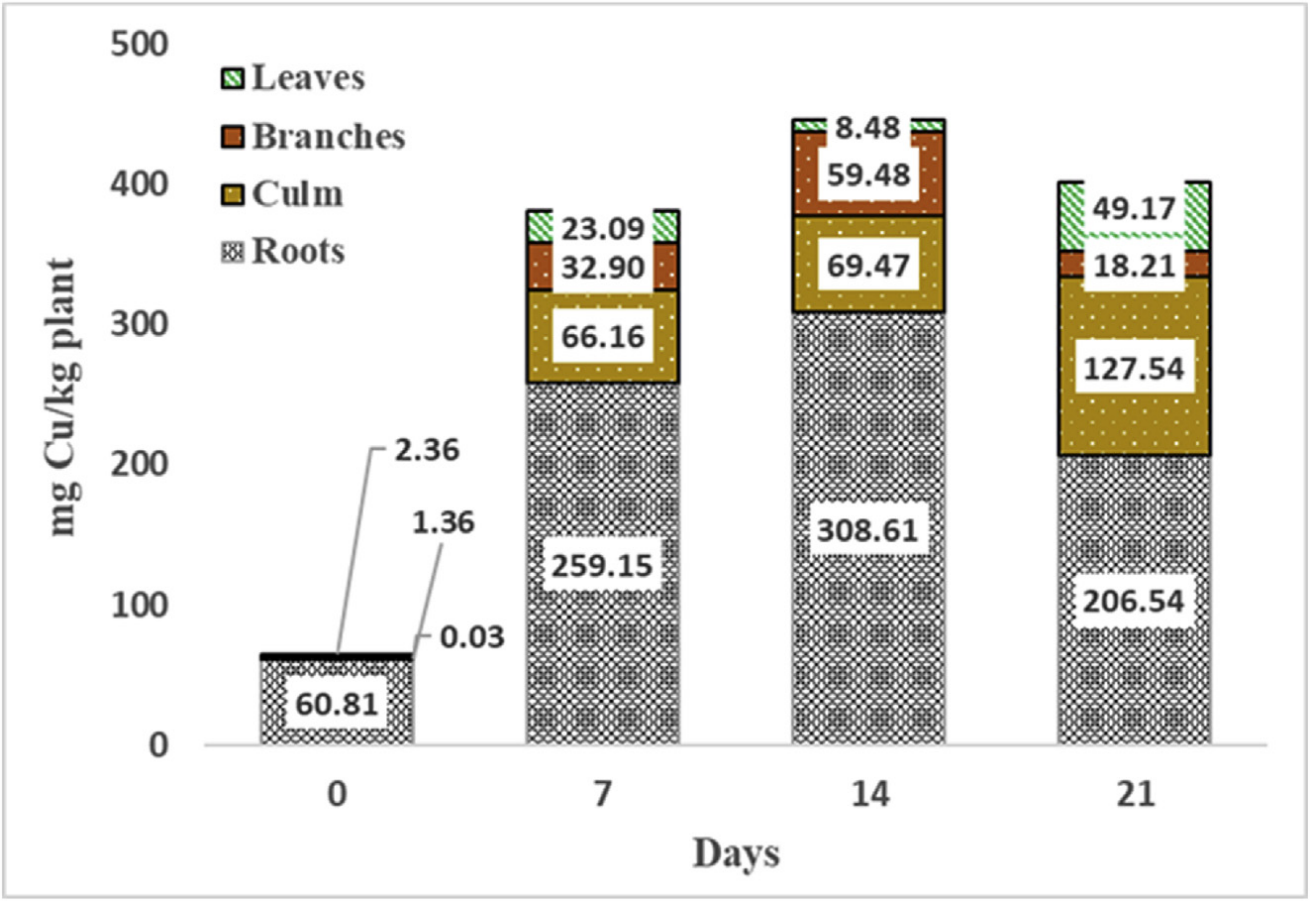
Copper accumulation in the roots, culm, branches and leaves for *D. asper.*

Fig. 2 shows the Cu concentration in the *D. asper* in different plants: roots, culm, branches and leaves. Generally, the concentration of Cu uptake within the plant increased as time progressed with the root system accumulating the largest concentration of Cu—which could indicate a phytostabilization potential. Most of the plant parts showed an increase in Cu in the first 14 days of the experiment, then on 21d, the Cu concentration in roots and branches started to decline. This behavior may signify the start of translocation of the contaminant to the other parts of the plant. Using the Cu concentrations at 21d, the translocation factors (TF) for the culm, branches and leaves were previously found to be 0.42, 0.063 and 0.17, respectively (Arcilla et al., 2017b). The culm demonstrated an increasing concentration of Cu at 21d implying that the immediate increase in the biomass of the bamboo plant is concentrated in the culm (about 80%). Another possible explanation for this increase is the culm size of the bamboo consisting mostly of sclerenchyma cells; hence, the translocation is slower than that of the Cu uptake to the roots, a phenomenon observed in the *Eucalyptus* species (A. Emamverdian et al., 2015).

Statistical analyses showed that the *F-*value and *p-*value for species selection were 4.05 and 0.0305, respectively, whereas the *F-*value and *p-*value for the varying concentration levels were 7.52 and 0.0025, respectively. The *F-*value for these two phases were higher than the critical value of *F* (*F*_*crit*_ = 3.403), which indicates that for Phase 1, different bamboo species uptake Cu differently; whereas for Phase 2, initial Cu concentration plays a vital role in Cu removal (accumulation). To further prove the significance of the data, the *p*-value was compared to *alpha*. Both *p*-values in the two phases were less than 0.05, which proves the significance among the species and initial Cu concentrations.

### 3.3 Kinetics of phytoremediation

**Fig. 3:**
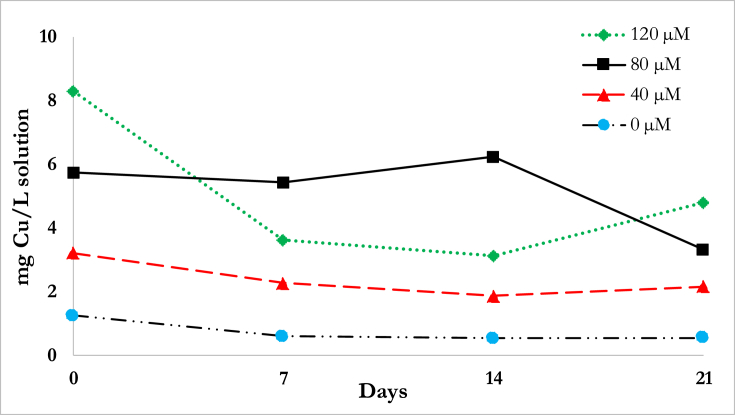
Cu concentration in the solution at varying initial Cu contaminations.

The kinetics of contaminant removal were obtained from the concentration of Cu in the nutrient solution during sampling times at 7, 14, and 21d for the control and the different treatments. Fig. 3 illustrates the Cu concentration in the nutrient solution for the different contaminations. In general, the concentration decreased with time except for the highest contamination at 120 μM. Defense mechanisms of the plant system could have prevented the entry of Cu while allowing the intake of other elements in the nutrient solution or water. Expulsion of excess Cu could have also occurred (Hall, 2002).

Using the rate of contaminant removal from the differential Eq. (3) at *n* = 0 and *n* = 1, the data obtained were linearized according to Table 3, which allowed for obtaining the kinetic parameters and R^2^ as shown in Table 5. Both 0^th^ and 1^st^ order models showed poor fit to the experimental data. Other published studies often show good fits for the contaminant removal with *R*^*2*^ values greater than 0.9. A phytoremediation kinetics study by Cui and Schröder (2016) for a duration of 28 days revealed that the first order disappearance of metformin at initial concentrations from 50 to 250 μM using *T. latifolia* had good fits at *R*^*2*^ values ranging from 0.912 to 0.985. Two factors that could have attributed to the deviation from the literature are: (1) the nature of the contaminant where PTEs could behave differently than organic compounds. Metformin could have decreased in concentration due to degradation in addition to uptake, whereas in this study, Cu disappearance was only assumed to be due to uptake, possibly explaining why the removal efficiencies for metformin were higher (Cui and Schröder, 2016); and (2) the larger solution uptake of *D. asper*. Depending on the root system, the uptake of the solution may be different. For smaller roots, the volume change could be negligible, so it is assumed that the concentration change is only due to contaminant removal, as in the case of the phytoremediation of Cu and Zn by *E. crassipes* (Naaz et al., 2013).

**Table 5:** R^2^ and kinetic parameters for the 0^th^ and 1^st^ order rate of Cu disappearance.

Order	*n* = 0		*n* = 1		Percent Removal (*C*_*S*_*/C*_*i*_) at *t* = 21 days
Concentration	*R*^*2*^	*k* (mg-L^−1^-d^−1^)	*R*^*2*^	*k* (d^−1^)	
*Control*	0.663	0.031	0.692	0.037	43.24%
40 μM	0.624	0.051	0.606	0.020	67.22%
80 μM	0.419	0.092	0.465	0.021	58.04%
120 μM	0.369	0.157	0.287	0.026	57.90%

Note: Control – without addition of Cu contaminant.

The low *R*^*2*^ values from Table 5 may come from the errors due to the large volume uptake of the *D. asper*, thus causing deviations in the concentration where the amount of Cu uptake is no longer proportional to the nutrient absorption. The *D. asper* has a large roots system with the volume uptake on average at 286.14 ml.

**Fig. 4:**
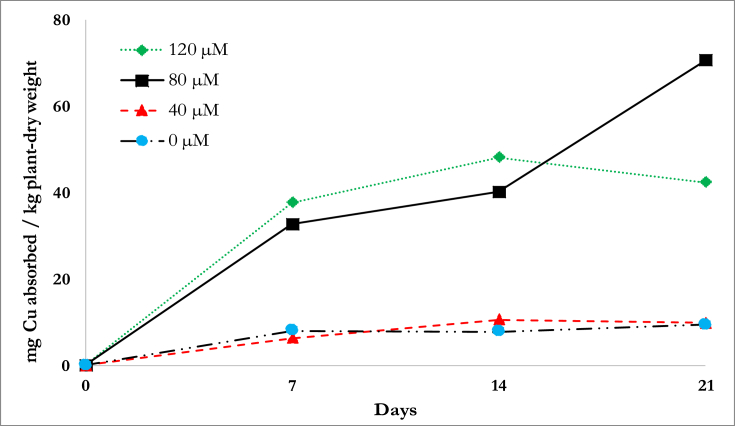
Copper uptake at varying initial copper concentrations.

Fig. 4 illustrates the amount of Cu uptake per plant on a mass basis. Using the rate equation in Eq. (4) at n = 0 & n = 1, R^2^ and kinetic parameters were obtained as shown in Table 6. The kinetic fit for the 0^th^ order uptake generally produced higher R^2^ compared to the 1^st^ order. The rate constant, *k*, was highest at the 80 μM for both 0^th^ and 1^st^ order model which means that the rate uptake from the solution is highest at this concentration, supporting the results of Prasad et al. (Prasad and Freitas, 2003).

**Table 6:** R^2^ and kinetic parameters for the 0^th^ and 1^st^ order rate of Cu uptake.

Order	n = 0		n = 1	
Concentration	*R*^*2*^	k (mg-kg^−1^-d^−1^)	*R*^*2*^	k (d^−1^)
*Control*	0.723	0.401	0.780	1.988
40 μM	0.818	0.484	0.728	1.167
80 μM	0.954	3.136	0.948	1.410
120 μM	0.666	1.970	0.631	2.175

Note: Control – without addition of Cu contaminant.

**Fig. 5:**
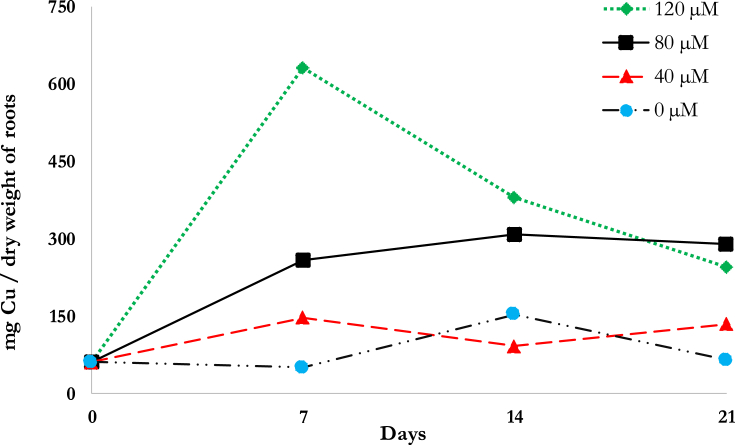
Accumulation within the roots of *D. asper* at varying initial Cu concentrations.

The root system plays an important part in phytoremediation because it is where initial contact with the contaminant happens. The accumulation of Cu in the roots of *D. asper* is shown in Fig. 5. The trends in the Cu accumulation indicate that the concentrations within the roots were stable at 40 μM. Compared to the higher concentrations, the Cu uptake pattern for both the control and 40 μM were similar. Because 40 μM is within the tolerable range of Cu concentrations for plants, it is possible that the roots behaved as if it were stabilizing the Cu concentration within its cells, translocating when necessary. The Cu uptake at 80 μM reached its peak on 14d then slightly declined (5.26%) after that. This could possibly due to the plant's roots having reached their saturation point for the uptake of Cu. At 120 μM, the roots had their highest uptake on 7d, but the Cu concentrations declined thereafter such that on 21d, the concentration of Cu in the roots was lower than in the 80 μM-contaminated plants. The peak at 120 μM, which was also the highest Cu accumulation achieved in the study could be due to the initial uptake by the roots of an abundant supply of Cu in their proximity. The decline of Cu in the succeeding days could be due to its translocation to other plant parts such as the culm and leaves. It is also possible that the roots are restricting the entry of Cu into its cells to prevent further damage (Benyo et al., 2016). At 80 μM, the slight decrease in Cu accumulation on 21d could indicate that translocation occurred. Similarly, at 120 μM, the increase in Cu uptake on 7d followed by a drastic decline on 14d and 21d suggests that translocation occurred. The BCF based on Cu accumulation on 21d for 40, 80 and 120 μM were 41.86, 50.57 and 29.63, respectively.

The amount of Cu accumulated within the roots was used in the Michaelis–Menten equation for the kinetics study. The linearized form as shown is Table 3 was used to fit the data on 7, 14, and 21d. The kinetic parameters and *R*^*2*^ values are shown in Table 7 where the data from the earliest sampling time had the best fit. The Michaelis–Menten model assumes a constant concentration solution and experiments for this model are typically carried out in short periods of time to prevent depletion of solution. The poor fit for 14d can be traced back to Fig. 5 where the oscillation of the 40 μM solution had an accumulation value lower than that of the control, thus deviating from the expected linear trend (i.e., higher initial concentration resulted in higher Cu accumulation).

**Table 7:** R^2^ and kinetic parameters for the Michaelis–Menten equation.

Kinetic Parameters	Day of Sampling		
	7	14	21
*V*_*max*_ (mg Cu-kg plant^−1^-day^−1^)	66.26	13.91	13.68
*K*_*M*_ (mg Cu-L^−1^)	4.87	0.23	2.06
*R*^*2*^	0.97	0.05	0.95

This study provides a valuable insight into the absorption and translocation of Cu in *D. asper* using hydroponic culture. The results reveal that *D. asper* can tolerate high Cu concentrations, which bodes well for its potential utilization in phytoremediation strategies.

A number of studies have reported that Cu accumulates more in the roots than in aboveground parts of other grass species (Ait Ali et al., 2002; Ouzounidou et al., 1995) and in the bamboo *Gigantochloa* sp. “Malay Dwarf” (Blanche et al., 2013). A previous study (Babatunde et al., 2009) suggested that bamboo could be effective in the adsorbent removal of Cu in wastewater. In that case, *D. asper* could potentially be effective in two types of phytoremediation strategies: rhizofiltration and phytostabilization. The former involves filtering heavy metals from water onto the root system (Zhang et al., 2010), and the latter involves reducing heavy metal concentrations in soil by decreasing their mobility or bioavailability (Suman et al., 2018). It is also possible that because of their large root biomass, *D. asper* and other bamboo species can also play as erosion control agents near contaminated bodies of water. Furthermore, because it grows very quickly and multiplies rapidly, it is also possible for bamboo to lower the Cu concentration in contaminated water or soil through internal utilization as Cu is an essential element for plant growth.

The use of soil-based media would have been more representative of natural, field conditions and thus, a better test for phytoremediation ability. But in this study, hydroponics made it easier to select the best Cu-uptaking bamboo species and allowed for a better understanding of the absorption kinetics of Cu in *D. asper*. Future studies using pot-scale experiments or under contaminated field conditions are warranted to further verify the initial findings of this study.

## 4 Conclusions

*D. asper* exhibited phytoremediation potential as evident by the increasing copper uptake using hydroponics solution. Within the plant tissues, roots accumulated the highest concentration of Cu indicating a phytostabilization potential. The kinetics of Cu uptake best fitted with the 0^th^ order at *R*^*2*^ value of 0.954 for initial Cu concentration of 80 μM, with the rate constant generally increasing at 3.36 mg Cu/kg plant mass-day. Kinetic studies performed for Cu accumulation modelled using the Michaelis–Menten equation showed the best fit on 7d with an *R*^*2*^ of 0.970, *K*_*M*_ = 4.87 mg/L and *V*_*max*_ = 66.26 mg Cu-kg^−1^-day^−1^.

Given the results of the present work, it is highly recommended to test the kinetics of Cu uptake at shorter time intervals for longer experiment periods, *e.g.* 1, 3, 5d interval for a period of 2 months. Also, it is recommended to evaluate the phytoremediation potential of other locally versatile bamboo species including *B. vulgaris, Gigantochloa levis,* and *Schizotachyum lumampao* in both hydroponics and soil-based media.

## Declarations

### Author contribution statement

Jennivee Chua, Jessa Marie Banua, Ivan Lawrence Arcilla: Performed the experiments; Analyzed and interpreted the data; Wrote the paper.

Aileen Orbecido: Analyzed and interpreted the data; Contributed reagents, materials, analysis tools or data; Wrote the paper.

Maria Ellenita De Castro: Analyzed and interpreted the data; Wrote the paper.

Nadine Ledesma, Lawrence Belo: Conceived and designed the experiments; Analyzed and interpreted the data; Contributed reagents, materials, analysis tools or data; Wrote the paper.

Custer Deocaris, Cynthia Madrazo: Conceived and designed the experiments; Analyzed and interpreted the data; Wrote the paper.

### Funding statement

This work was supported by the University Research Coordination Office (URCO) of De La Salle University for the Interdisciplinary Research funding (12 IR S 3TAY15-3TAY16).

### Competing interest statement

The authors declare no conflict of interest.

### Additional information

No additional information is available for this paper.
